# Supplementary material for: Co-Culturing Microalgae with Roseobacter Clade Bacteria as a Strategy for Vibrionaceae Control in Microalgae-Enriched Artemia
Source: Microorganisms. 2023 Nov 6;11(11):2715. doi: 10.3390/microorganisms11112715 (PMC10673095; doi:10.3390/microorganisms11112715)
Supplement: Supplementary file 1 [file microorganisms-11-02715-s001.zip › microorganisms-2632337-supplementary.pdf]

SUPPLEMENTARY MATERIALS

Co-Culturing Microalgae with *Roseobacter* Clade Bacteria as a Strategy for *Vibrionaceae* Control in Microalgae-Enriched *Artemia*

José Pintado [1,*], Patricia Ruiz [1], Gonzalo Del Olmo [1] and Pavlos Makridis [2]

[1] Marine Ecology and Resources Group, Institute of Marine Research (IIM-CSIC), 36208 Vigo, Spain; patriciaruiz@iim.csic.es (P.R.); gdelolmo@iim.csic.es (G.D.O.)
[2] Department of Biology, University of Patras, 26504 Rio Achaias, Greece; makridis@upatras.gr
* Correspondence: pintado@iim.csic.es

SUPPLEMENTARY FIGURES

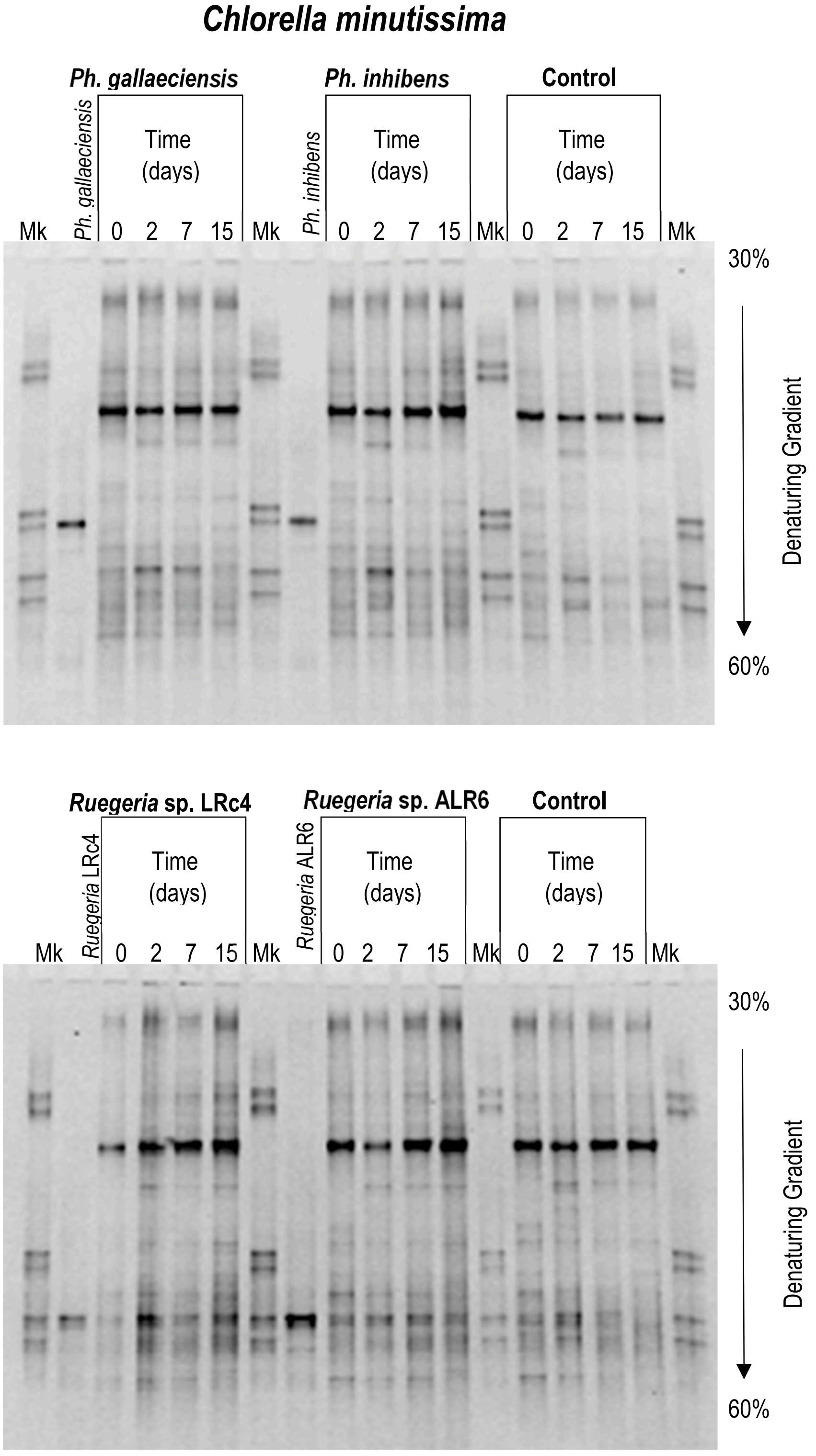

Figure S1: DGGE profiles of the bacterial communities present in the mixed cultures of the algae *Chlorella minutissima* and the bacteria *Phaeobacter gallaeciensis, Phaeobacter inhibens, Ruegeria* sp. LRc4 and *Ruegeria* sp. ALR6 and Control cultures with no addition of bacteria. Positive controls of the inoculated bacteria are included. Mk: marker.

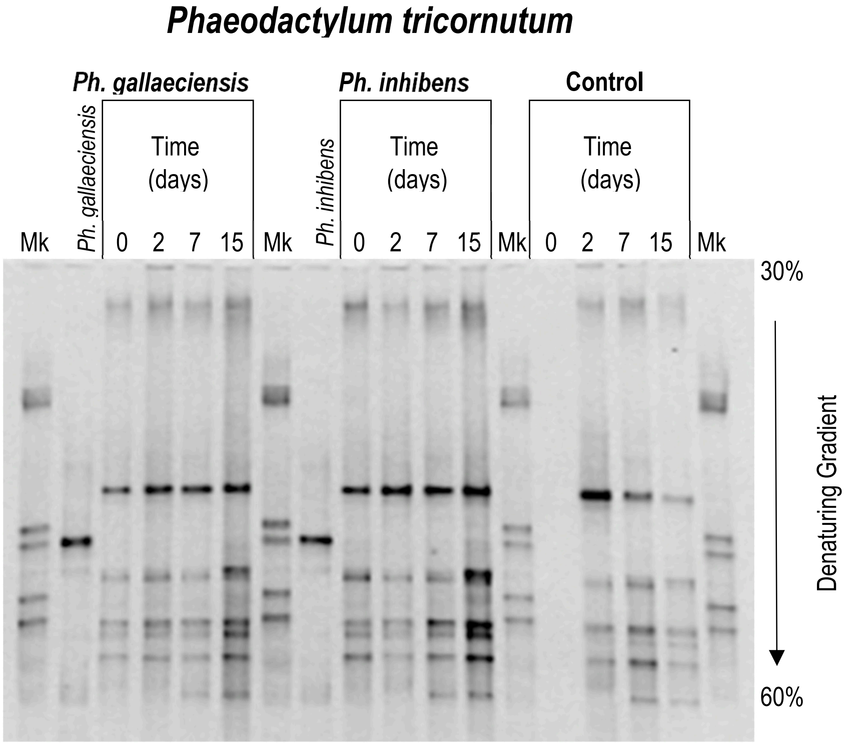

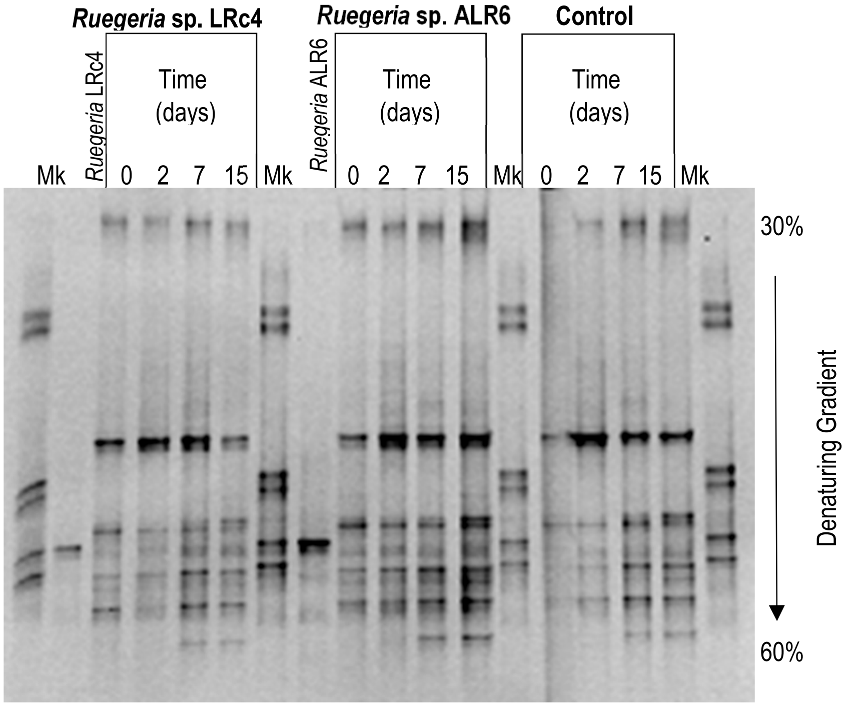

Figure S2: DGGE profiles of the bacterial communities present in the mixed cultures of the algae *Phaeodactylum tricornutum* and the bacteria *Phaeobacter gallaeciensis*, *Phaeobacter inhibens*, *Ruegeria* sp. LRc4 and *Ruegeria* sp. ALR6 and Control cultures with no addition of bacteria. Positive controls of the inoculated bacteria are included. Mk: marker.

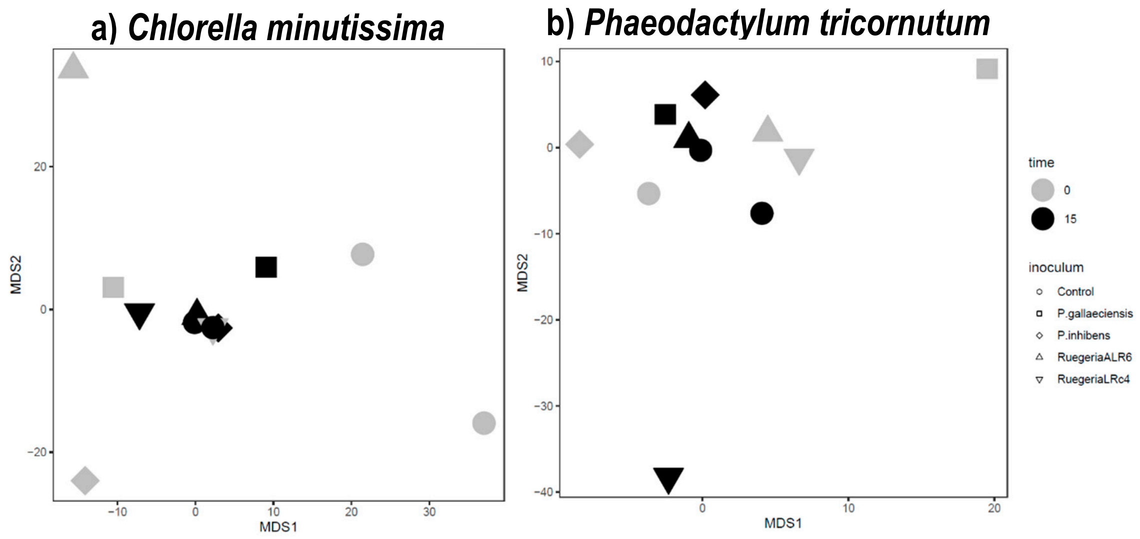

Figure S3: Multidimensional Scaling (MDS) plot of bacterial communities from samples of the mixed cultures of the algae *Chlorella minutissima* (a) and *Phaeodactylum tricornutum* (b) with the bacteria *Phaeobacter gallaeciensis*, *Phaeobacter inhibens*, *Ruegeria* LRc4 and *Ruegeria* ALR6, and Control cultures of algae with no addition of bacteria.

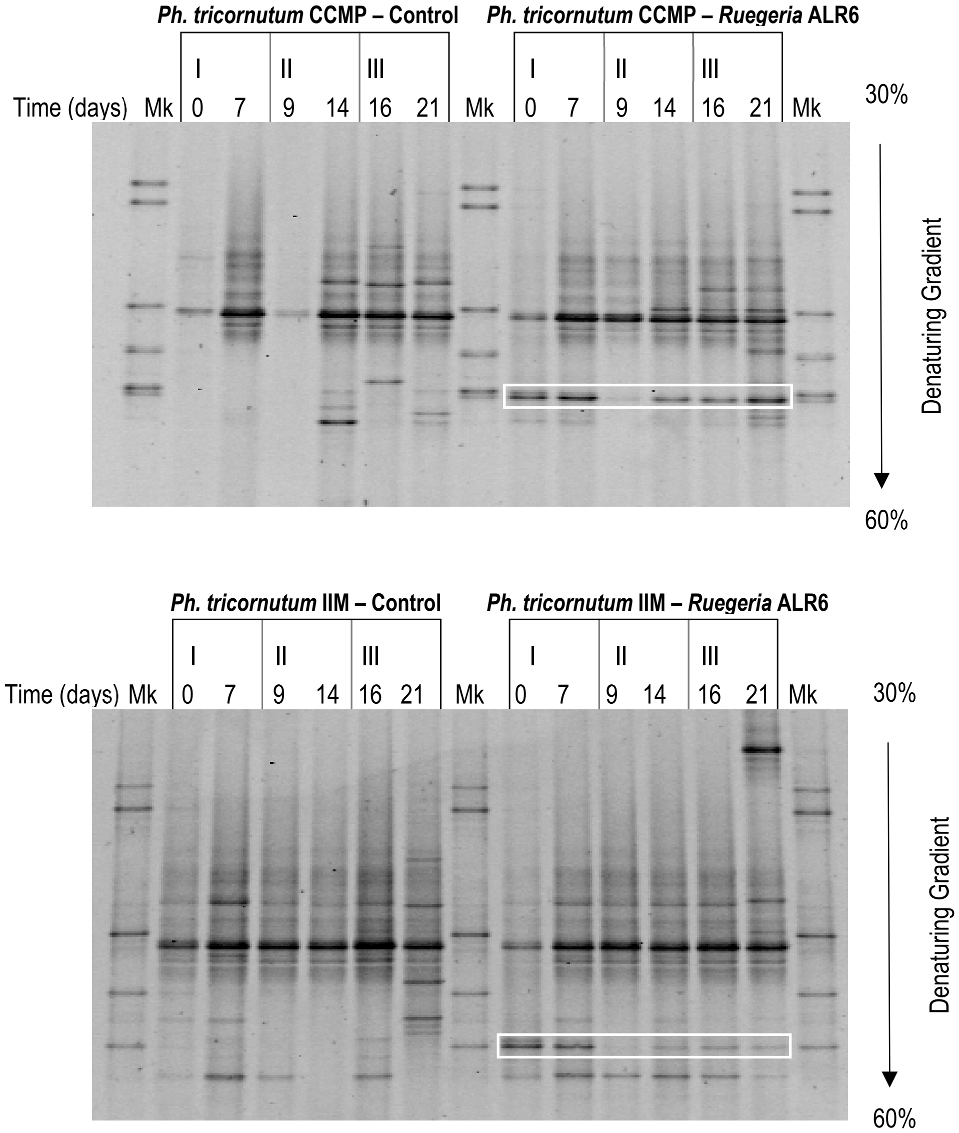

Figure S4: DGGE profiles of the bacterial communities present in *Phaeodactylum tricornutum* cultures of axenic (CCMP) or non-axenic (IIM) strains inoculated with *Ruegeria* sp. ALR6 or not inoculated (Control), scaled-up from (I) 100 ml flask to (II) 5 L flask and to (III) 50 L bags. Outlined in white the band corresponding to *Ruegeria* sp. ALR6.

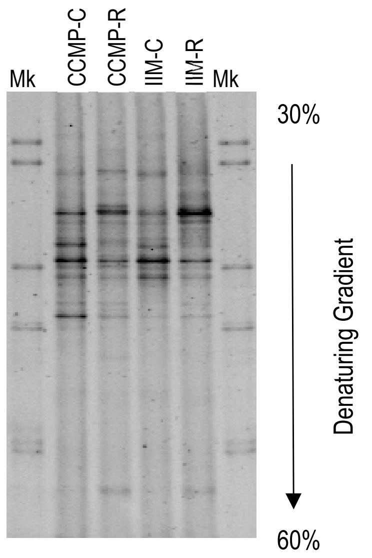

Figure S5: DGGE profiles of the bacterial communities present in *Artemia* enriched 48 h with the algae *Phaeodactylum tricornutum* strains CCMP and IIM, with introduction of bacteria *Ruegeria* sp. ALR6 (CCMP-R and IIM-R) or without introduced bacteria (Control, CCMP-C and IIM-C). Mk: marker.

SUPPLEMENTARY TABLES

Table S1: Confidence Interval (C.I.) comparations between the different amount of bacteria co-cultivated with different microalgae. It is shown pairwise comparisons between the C.I. of the counts of the different bacteria (*Phaeobacter gallaeciensis, Phaeobacter inhibens, Ruegeria* sp. ALR6 and *Ruegeria* sp. LRc4) co-cultivated with different microalgae (*Chlorella minutissima* and *Phaeodactylum tricrnutum*) at time 15 of the culture. It is only compared the C.I. of the same bacteria counts in the presence of the different microalgae studied. The comparisons are based on the overlapping of the C.I. Analysis made in Rstudio version 4.3.1.

Bacterial	Time	Microalgae	CI	Comparisons
Phaeobacter_gallaeciensis	15	Chlorella_minutissima	3.99 - 4.10	a
		Phaeodactylum_tricornutum	3.89 - 5.14	a
Phaeobacter_inhibens	15	Chlorella_minutissima	4.55 - 5.02	a
		Phaeodactylum_tricornutum	4.60 - 6.31	a
Ruegeria_ALR6	15	Chlorella_minutissima	3.71 - 4.91	a
		Phaeodactylum_tricornutum	6.28 - 6.63	b
Ruegeria_LRc4	15	Chlorella_minutissima	4.36 - 5.04	a
		Phaeodactylum_tricornutum	5.39 - 5.56	b

Table S2: Confidence Interval (C.I.) comparations between the different amount of *Ruegeria* sp. ALR6 co-cultivated with *P. tricornutum* (CCMP: axenic and IIM: non-axenic) at time 7, 14 and 21, the lasts stages of the scale-up experiment. The comparisons are based on the overlapping of the C.I. Analysis made in Rstudio version 4.3.1.

Bacteria	Time	Microalgae	C.I.	Comparisons
Ruegeria_ALR6	7	Phaeodactylum_CCMP	6.83 - 6.86	a
		Phaeodactylum_IIM	6.52 - 7.07	a
	14	Phaeodactylum_CCMP	5.65 - 7.20	a
		Phaeodactylum_IIM	5.68 - 6.26	a
	21	Phaeodactylum_CCMP	6.20 - 6.38	a
		Phaeodactylum_IIM	5.05 - 5.42	b

Table S3: Confidence Interval (C.I.) comparations between the different amount of Vibrionacea counts in *Artemia* during the enrichment with *P. tricornutum* from initially axenic (CCMP) or initially non-axenic (IIM) microalgae strains co-cultured with *Ruegeria* sp. ALR6 or without (Control). The comparisons are based on the overlapping of the C.I. Analysis made in Rstudio version 4.3.1.

Microalgae	Time	Bacteria	C.I.	Comparisons
Phaeodactylum_CCMP	4	Control	6.59 - 7.45	a
		Ruegeria_ALR6	4.56 - 4.60	b
Phaeodactylum_IIM	4	Control	6.39 - 6.63	a
		Ruegeria_ALR6	5.59 - 5.68	b
